# Skin Application of Inflammasome Inhibitor MCC950 Prevents Psoriasis-like Cutaneous Manifestations and Associated Systemic Inflammation

**DOI:** 10.1007/s10753-026-02535-1

**Published:** 2026-06-04

**Authors:** Chiara Terranova, Alex Spitilli, Alisia Camporeale, Francesca Nigro, Federica Brugnoli, Silvia Grassilli, Valeria Bertagnolo, Fabio Casciano, Hasan Ayaz, Enrico Tassani, Chiara Ruzza, Claudio Trapella, Francesca Granucci, Davide Ferrari, Paola Secchiero, Eva Reali

**Affiliations:** 1 Department of Translational Medicine, University of Ferrara, Ferrara, Italy; 2 Department of Life Sciences and Biotechnology, University of Ferrara, Ferrara, Italy; 3 Department of Environmental and Prevention Sciences and LTTA Centre, University of Ferrara, Ferrara, Italy; 4 Department of Biotechnology and Biosciences, University of Milano-Bicocca, Milan, Italy; 5 Department of Neurosciences and Rehabilitation, University of Ferrara, Ferrara, Italy; 6 Department of Chemical Pharmaceutical and Agricultural Sciences, University of Ferrara, Ferrara, Italy

**Keywords:** NLRP3 inflammasome, Skin inflammation, Upstream targeting, T-cell trafficking, Systemic effects

## Abstract

Psoriasis is a chronic, systemic inflammatory disease associated with cardiovascular and metabolic comorbidities. It is driven by a sustained immune response in the skin, orchestrated by keratinocytes and both innate and adaptive immune cells. The initial phase of the pathology remains largely unclear. It likely involves the activation of dendritic cells by stress signals from keratinocytes with the NLRP3 inflammasome potentially acting as a key innate sensor. Here we investigate the effects of the topical administration of the selective NLRP3 inhibitor MCC950, in the imiquimod-induced psoriasis-like inflammation model with the aim to achieve the inhibition of both cutaneous and systemic manifestations. Topical MCC950 effectively suppressed NLRP3 inflammasome activation by preventing pro-caspase-1 cleavage and generation of the active p20 subunit in the skin. Gene expression analysis showed that inhibition of NLRP3 suppressed the expression of psoriasis hallmark genes, including *Il17*, *Tnf*, *S100a8/a9* and *Il1b*. Furthermore, network analysis identified NLRP3 as a central hub in the psoriasis-associated inflammatory module, supporting the concept that inflammasome inhibition dampens multiple downstream inflammatory pathways. In psoriasis-like condition, MCC950 reduced the skin infiltration of CD3^+^ T cells, CD11b^+^ myeloid and CD11c^+^ dendritic cells. Notably, MCC950 also prevented the systemic effects of psoriasis-like inflammation. Analysis in the spleen shows that it effectively prevented the phenotypic changes induced by psoriasis-like inflammation including the increased frequency of IL-17 A^+^ T cells. Finally, MCC950 prevented the systemic increase of serum IL-6. These findings highlight that upstream targeting of the NLRP3 inflammasome by MCC950 represents a promising strategy to inhibit both initiation and the systemic spread of psoriasis-like inflammation.

**Supplementary Information:**

The online version contains supplementary material available at 10.1007/s10753-026-02535-1.

## Introduction

Psoriasis is a chronic, inflammatory disease characterized by cutaneous erythematous, scaly plaques affecting about 2% of the population worldwide [1]. It is now recognized that psoriasis is not only a skin disease, but rather a systemic disorder, with both cutaneous and extracutaneous manifestations, associated with comorbidities such as metabolic syndrome and cardiovascular disease [1–4]. Pathology develops from a series of still poorly characterized events likely involving the interaction between keratinocytes and adaptive and innate immune cells, which orchestrate a sustained inflammatory response in the psoriatic plaques [5–7].

A key feature of the initial phase of the disease is the activation of dendritic cells (DCs) by mechanisms that are likely to involve stress and damage signals from keratinocytes, in which inflammasome complexes could play a crucial role [2, 5, 8]. Inflammasome multiprotein cytosolic structures, comprising a specific sensor protein, the ASC adaptor, and caspase-1, detect pathogen/damage-associated molecular patterns to trigger the release of interleukin-1 beta (IL-1β) and IL-18 [9, 10]. Among these, the NLRP3 has emerged as a critical driver implicated in a broad spectrum of diseases [11–14]. Pathological conditions characterized by mitochondrial reactive oxygen species (ROS) indeed promote NLRP3 activation [15]. NLRP3 is expressed by various cell types including myeloid cells and epithelial barrier cells, such as keratinocytes [11]. Its presence in the skin has been reported together with AIM2 and NLRP1 inflammasomes [16]. Genetic studies on different patient cohorts have identified multiple *NLRP3* single nucleotide polymorphisms (SNPs) as significant risk variants for psoriasis susceptibility. rs10754558 has also emerged as a prognostic biomarker, predicting non-response to systemic therapy [17–19].

For these reasons, the upstream targeting of innate immune sensors like NLRP3 may offer a promising therapeutic strategy [10, 14, 20, 21], as it could reduce both the initial phase of psoriasis and the signals necessary for the amplification of pathogenic T cell responses [22].The small molecule inhibitor MCC950 has demonstrated selective blockade of inflammasome activation preventing the assembly of the NLRP3 complex and inhibiting IL-1β and IL-18 maturation [23]. Initially developed for systemic inflammatory diseases, in the psoriasis mouse model MCC950 reduced skin symptoms upon systemic/intragastric administration. However, the high systemic toxicity has strongly limited its application as a therapeutic agent [24–27].

Here we want to explore the effects of topical application of MCC950 in the context of psoriasis with the aim of limiting both skin and systemic manifestations.

## Results

### NLRP3 Inhibitor MCC950 Prevents Psoriasis-like Inflammation and Preserved Histological Skin Architecture

**Fig. 1:**
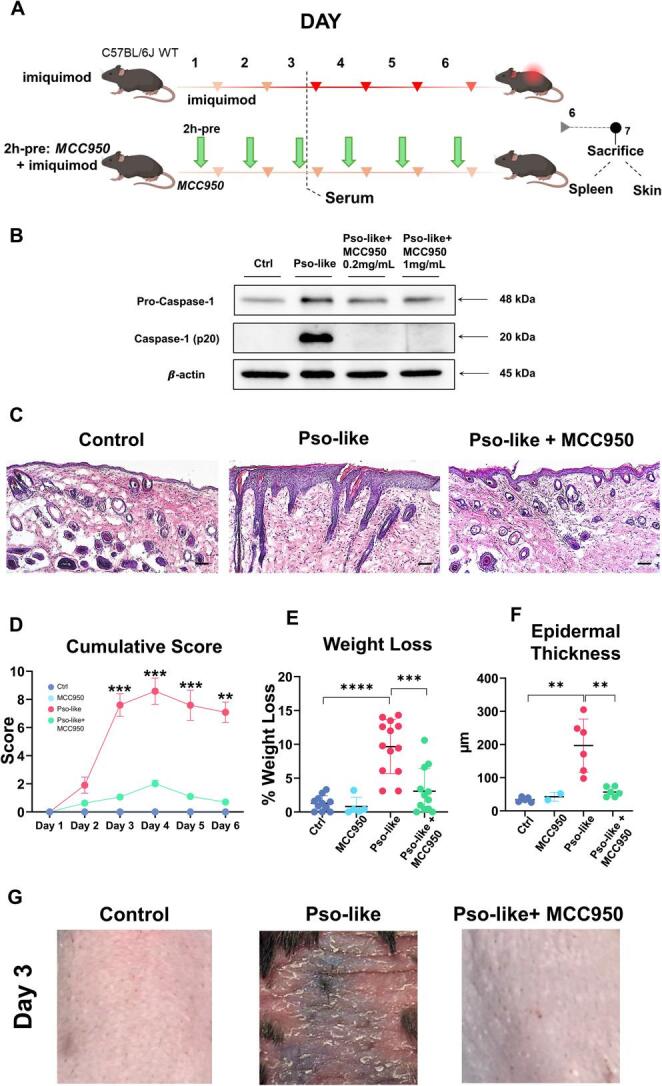
MCC950 inflammasome inhibitor prevents clinical and histological features of psoriasis-like inflammation. (**A**) Experimental Design: C57BL/6J mice received imiquimod applied topically to the shaved back skin and ears for 6 days. MCC950 was applied topically 2 h before the imiquimod application. Serum was collected on day 3, while on day 8 mice were euthanized, and spleen and skin tissues were harvested for further analysis. (**B**) Representative Western Blot analysis of Pro-Caspase-1 and cleaved Caspase-1 (p20) in skin lysates obtained from control (Ctrl, *n* = 3), psoriasis-like (Pso-like, *n* = 3) and psoriasis-like mice pre-treated with MCC950 at 0.2 mg/mL (Pso-like+MCC950 0.2 mg/mL, *n* = 3) and 1 mg/mL (Pso-like+MCC950 1 mg/mL, *n* = 3). β-actin was blotted as an internal control for equivalence of loaded proteins. The figure shows one representative experiment out of three independent experiments performed. (**C**) Histological analysis of frozen skin sections from the control, psoriasis-like and psoriasis-like pre-treated with MCC950, stained with Hematoxylin and Eosin (H&E). One representative image per group. Scale bar = 100 μm, magnification, 10x. (**D**) Daily monitoring of redness, scaling and thickness is represented as cumulative clinical score graph (assessed on a scale from 0 to 12) as an increasing curve in control (ctrl, *n* = 11), MCC950 alone (MCC950, *n* = 5), psoriasis-like (pso-like, *n* = 13), psoriasis-like MCC950 pre-treated (pso-like+MCC950, *n* = 12) mice. Data are presented as mean ± SEM. (**E**) The plots represent the percentage of body weight loss expressed as the percentage change from baseline (day 1) to day 6 across the different groups. Data is presented as mean ± SD of values obtained from individual mice. (**F**) The plots represent the epidermal thickness (µm) in the different experimental groups. Data are presented as mean ± SEM. Two independent sections derived from two distinct skin regions were evaluated in control (ctrl, *n* = 5), MCC950 alone (MCC950, *n* = 2), psoriasis-like (pso-like, *n* = 6), psoriasis-like MCC950 pre-treated (pso-like+MCC950, *n* = 6) mice. Significance levels: *p < 0.05, **p < 0.01, ***p < 0.001, ****p < 0.0001. (**G**) Clinical signs of psoriasis-like inflammation in three representative mice belonging to control (ctrl), psoriasis-like (pso-like), psoriasis-like MCC950 pre-treated (pso-like+MCC950) group

To explore the effect of the NLRP3 inhibitor MCC950 on the development of psoriasis-like inflammation, mice were treated according to the protocol originally described by van der Fits et al. (Fig. 1A) [28]. To investigate whether topical application of MCC950 was capable of effectively inhibiting skin inflammasome activation, total and cleaved caspase-1 levels were assessed in tissue lysates by Western blot analysis. Cleaved caspase-1 p20 was detected only in skin samples from psoriasis-like inflammation and was undetectable in mice pretreated with MCC950 at both concentrations 0.2 and 1 mg/mL (Fig. 1B). Total pro-caspase-1 was moderately increased in samples from mice with psoriasis-like inflammation compared to control, with minimal differences with MCC950 pretreated mice. Clinical evaluation was performed by daily scoring of erythema, thickness and scaling. As previously reported, imiquimod-treated mice exhibited hallmark features of psoriasiform dermatitis (Fig. 1C-G). Pretreatment with MCC950 (1 mg/mL) prevented these clinical signs (Fig. 1C-G). Psoriasis-like mice showed systemic manifestations, such as a 9.6% weight loss compared to controls (Fig. 1E), fully reverted by MCC950 pretreatment.

Histological analyses were performed with H&E to evaluate epidermal structure and dermal inflammatory infiltrates. The data showed increased epidermal thickness, hyperkeratosis and parakeratosis, with elongated epidermis and dermal inflammatory infiltrates (Fig. 1C). In contrast, MCC950-pretreated mice showed reduced inflammatory features (Fig. 1C-G). Epidermal architecture was largely preserved, with minimal hyperplasia and reduced dermal infiltrate (Fig. 1C). These results indicate that topical MCC950 significantly inhibits imiquimod-induced histopathological changes.

### MCC950 Reduces T cells Infiltration in Psoriasiform Lesions

To characterize the inflammatory infiltrate in the different treatment groups, back skin sections (7 μm thickness) were analyzed by direct immunofluorescence.

**Fig. 2:**
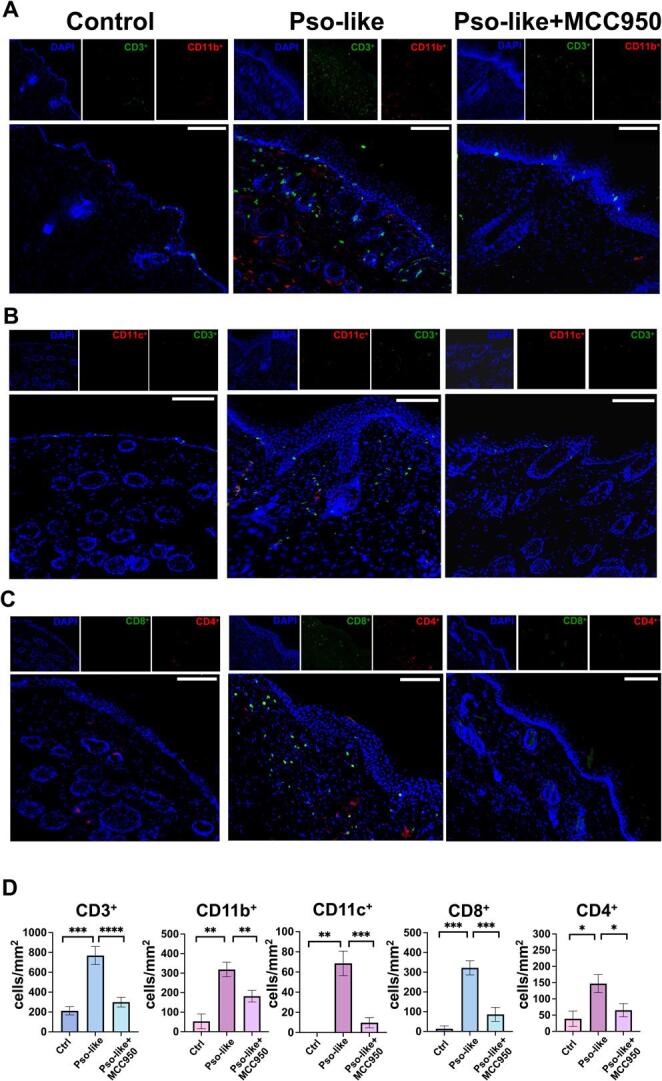
MCC950 inflammasome inhibitor prevents CD3^+^ and CD11b^+^ and CD11c^+^ cells accumulation in the skin in imiquimod-treated mice. (**A**) Representative immunofluorescence images showing DAPI (blue), CD3 (FITC, green), CD11b (PE, red) in control (ctrl), psoriasis-like (pso-like), psoriasis-like MCC950 pre-treated (pso-like+MCC950) mice. Back skin samples were collected and excised on day 8. Scale bar = 100 μm, magnification, 20x. (**B**) Representative immunofluorescence images showing DAPI (blue), CD3 (FITC, green), CD11c (PE, red) in control, psoriasis-like, psoriasis-like MCC950-pre-treated mice. Back skin samples were collected and excised on day 8. Scale bar = 100 μm, magnification, 20x. (**C**) Representative immunofluorescence images showing DAPI (blue), CD8 (FITC, green), CD4 (PE, red) in control, psoriasis-like, psoriasis-like MCC950-pre-treated mice. Back skin samples were collected and excised on day 8. Scale bar = 100 μm, magnification, 20x. (**D**) CD3⁺, CD11b⁺, CD8^+^, CD4^+^ and CD11c^+^ cells densities were calculated as the number of positive cells per unit area (cells/mm²) within each ROI. For each section, the mean value of two regions of interest (ROIs) was used for analysis. Mice groups were analyzed (ctrl, *n* = 4), (pso-like, *n* = 5), (pso-like+MCC950, *n* = 5), and for each mouse, two independent cryosections derived from two distinct skin regions were evaluated. Data is presented as mean ± SEM. Significance levels: *p < 0.05, **p < 0.01, ***p < 0.001, ****p < 0.0001

In mice with psoriasis-like inflammation, marked CD3^+^ T cell infiltration was observed throughout both epidermis and dermis on day 8, in parallel with increased CD11b⁺ cell infiltration within the dermis (Fig. 2A).

By contrast, mice with psoriasis-like inflammation pretreated with MCC950 showed a marked reduction in CD3^+^ T and CD11b^+^ cell density in the skin (Fig. 2A, D) as compared to mice with psoriasis-like inflammation. Additionally, analysis of the CD11c⁺ cells showed a significant increase of DCs in psoriasis-like mice, which was inhibited by treatment with MCC950 (Fig. 2B, D). In addition, we observed accumulation of CD8^+^ T cells in the epidermis/papillary dermis and CD4^+^ T cells in the dermis in psoriasis-like conditions. Notably, topical MCC950 treatment prevented the accumulation of both CD8⁺ and CD4^+^ T cells and restored their levels to those comparable to control animals (Fig. 2C, D).

### Different MCC950 Treatment Schedules Consistently Downregulate Key Pathogenic Genes in Imiquimod-induced Skin Inflammation

To investigate the molecular effects of topical NLRP3 inhibition on psoriasiform inflammation, we assessed the expression of hallmark genes (Supplemental Table 1) in skin tissues using quantitative reverse transcription PCR (RT-qPCR).

**Fig. 3:**
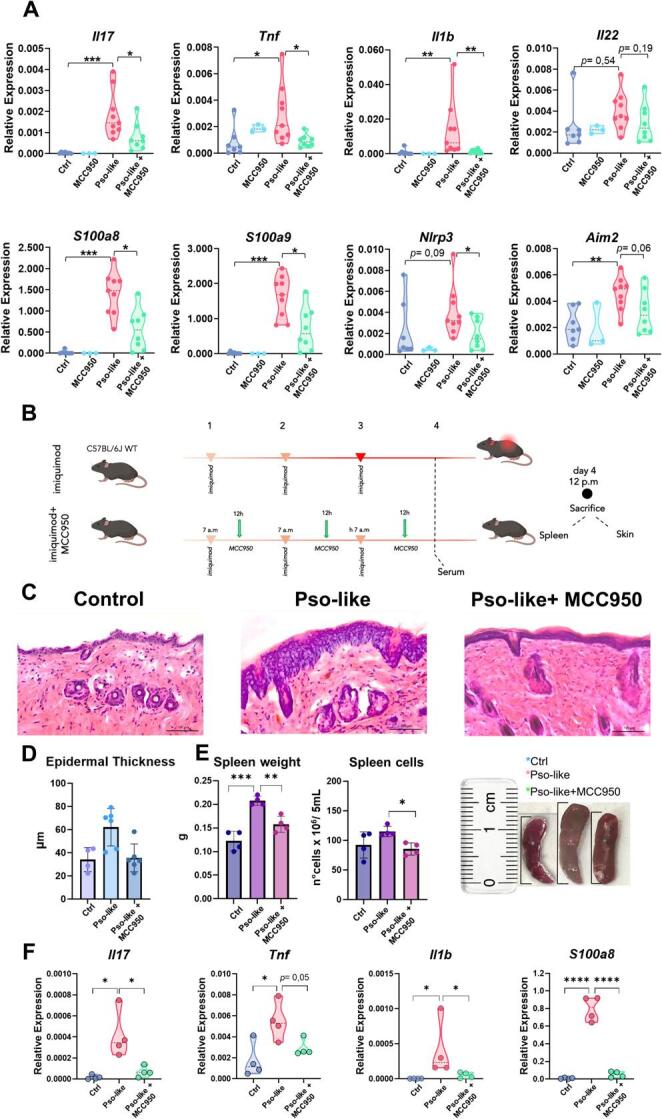
Different MCC950 treatment schedules consistently downregulate key pathogenic genes in imiquimod-induced skin inflammation. (**A**) Gene expression analysis by RT-qPCR was performed in skin samples from control (ctrl, *n* = 7), MCC950 alone (MCC950, *n* = 3), psoriasis-like (pso-like, *n* = 9), psoriasis-like MCC950 pre-treated (pso-like+MCC950, *n* = 8) mice. Relative expressions were calculated using the 2^−ΔCT with *Gapdh* as a reference gene. Violin plots showing the relative expression for each gene in different conditions are reported in the figure. (**B**) Experimental design for reverse treatment: C57BL/6J mice received imiquimod applied topically to the shaved back skin and ears for 4 days. Imiquimod was applied first, and residual cream was removed after 12 h before topical administration of MCC950. On day 4, mice were euthanized, and spleen, serum and skin tissues were collected for further analysis. (**C**) Histological analysis of frozen skin sections in the reverse treatment from the control, psoriasis-like and psoriasis-like treated with MCC950, stained with Hematoxylin and Eosin (H&E). One representative image per group. Scale bar = 100 μm, magnification, 20x. (**D**) The plots show the epidermal thickness (µm) in the different experimental groups relative to the reverse treatment schedule depicted in panel B. Data are presented as mean ± SD. Two independent sections derived from two distinct skin regions were evaluated in control (ctrl, *n* = 2), psoriasis-like (pso-like, *n* = 3), psoriasis-like MCC950 treated (pso-like+MCC950, *n* = 3) mice. (**E**) The plots show spleen weight and cells number from control (ctrl, *n* = 4), psoriasis-like (pso-like, *n* = 4), psoriasis-like MCC950 treated (pso-like+MCC950, *n* = 4) mice relative to the reverse treatment schedule depicted in panel B. The spleen weight is presented in (g). Data is presented as mean ± SD. Representative pictures of one spleen per group. (**F**) Gene expression analysis by RT-qPCR was performed in skin samples relative to the reverse treatment experiment depicted in panel B from control (ctrl, *n* = 4), psoriasis-like (pso-like, *n* = 4), psoriasis-like MCC950 treated (pso-like+MCC950, *n* = 4) mice. Relative expressions were calculated using the 2^−ΔCT with *Gapdh* as a reference gene. Violin plots showing the relative expression for each gene in different conditions are reported in the figure. Statistical analysis of the differences was performed by Mann–Whitney test or Student t-test according to the normality of the distribution. Significance levels: *p < 0.05, **p < 0.01, ***p < 0.001, ****p < 0.0001

As shown in Fig. 3A, *Il17*, *Tnf*,* Il1b*,* S100a8* and *S100a9* expression was significantly increased in the skin of mice with psoriasis-like inflammation compared to the control group. Skin administration of MCC950 resulted in a marked downregulation of their expression.

Analysis of inflammasome sensor expression in imiquimod-treated mice revealed a trend toward increased *Nlrp3* levels and a significant upregulation of *Aim2* (Fig. 3A). MCC950 pretreatment significantly inhibited *Nlrp3* expression and partially reduced *Aim2*, supporting coordinated inflammasome sensor regulation in inflamed skin.

No significant changes in vascularization induced by MCC950 were observed as detected by the expression of *Pecam1*, encoding the endothelial cell marker CD31 (Supplemental Fig. 2).

We also applied a reverse treatment schedule in which MCC950 was administered 12 h after imiquimod treatment (Fig. 3B). At the end of treatment, serum and skin samples were collected for histological analysis and RT-qPCR for assessment of key inflammatory genes. Notably, reversal in the treatment sequence showed comparable results, with epidermal thickness returning to levels comparable to controls following MCC950 treatment (Fig. 3C, D). In addition, mice with psoriasis-like inflammation exhibited a significant increase in spleen weight and total cell number, which was significantly reduced by MCC950 treatment (Fig. 3E). Gene expression analysis further confirmed this inflammatory profile (Fig. 3F). This data confirms that the anti-inflammatory effect of MCC950 on psoriasiform dermatitis occurs independently of treatment schedule.

### In Silico Gene Expression and Network-based Analysis Indicate NLRP3 as a Central Hub of Psoriasis-associated Inflammatory Pathways

**Fig. 4:**
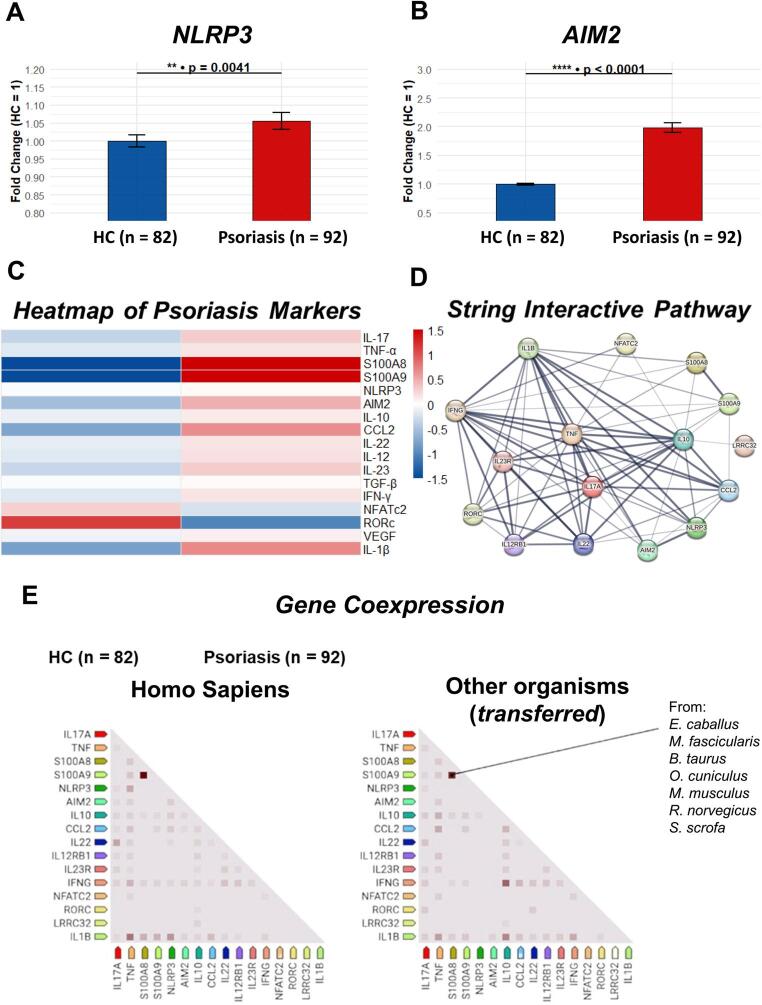
In silico gene expression and network-based analysis indicate NLRP3 as a central hub of psoriasis-associated inflammatory pathways. (**A**) Fold-change analysis of NLRP3 gene expression performed with R software on the human psoriasis dataset GSE54456. (**B**) Fold-change analysis of AIM2 gene expression performed with R software on the human psoriasis dataset GSE54456. (**C**) Heat-map analysis of human dataset GSE54456 with R software relative to psoriasis-related gene expression profiles in patients and control groups. (**D**) Confidence-based STRING pathway analysis (> 0.9) of human NLRP3 linked to its 15 high confidence interactors, edge thickness is proportional to the composite confidence score derived from seven independent evidence channels (neighborhood, gene-fusion, co-occurrence, co-expression, experimental/biochemical, database-curated, and text-mining). (**E**) The graphs show pairwise co-expression levels between NLRP3, and the 15 immune interactors as calculated by ProteomeHD. Intensity of red is proportional to the Pearson correlation coefficient (r) computed across human bulk-RNA-seq datasets and proteomics datasets runs downloaded from EMBL-EBI Expression Atlas from other 6 mammalian species (red = stronger positive co-expression; white = no detectable co-pattern)

It is noteworthy that analysis of patients’ dataset (GSE54456) [29] shows that both inflammasomes, *NLRP3* and *AIM2* genes, are upregulated in human psoriatic lesions as shown in Fig. 4A-C. In addition, to further understand the connection between inflammasome sensors NLRP3 and AIM2 with the network of inflammatory molecules in the human disease, we built a high-confidence protein-protein interaction network using STRING v12.0 (confidence ≥ 0.9) (Fig. 4D). We mainly focused on the differentially expressed markers of human pathology and of the mouse model: NLRP3, AIM2, IL-1β, IL-17 A, IL-22, TNF, S100A8 and S100A9. In this network, NLRP3 emerged as a central hub, connecting directly with 7 validated partners [30, 31].

These interactions form a tri-molecular platform that links NLRP3 with IL-1β and IL-18, and ultimately links to the NF-κB signaling pathway. Additionally, co-expression edges, derived from six mammalian datasets (Fig. 4E), show strong correlations: Pearson *r* = 0.75 between NLRP3 and TNF and *r* = 0.70 between NLRP3 and IL-17 A. Together, the interactome provides a clear topological explanation that NLRP3 inhibition can simultaneously suppress multiple key inflammatory molecules. Beyond the core inflammasome, the calgranulin heterodimer S100A8/S100A9 clustered closely with the NLRP3 hub. A strong co-expression and a gene co-occurrence edge indicate that these genes have been evolutionarily co-selected across 1,200 bacterial and vertebrate genomes. Similarly, a milder NLRP3-AIM2 co-expression confirms data obtained in the psoriasis mouse model.

### MCC950 Pretreatment Reverts Spleen Phenotypic Variations and Restores IL-6 Serum Levels in Psoriasis-like Inflammation

To explore the systemic immunological impact of skin NLRP3 inhibition in psoriasiform inflammation, we performed immunophenotyping of splenocytes by flow cytometry.

**Fig. 5:**
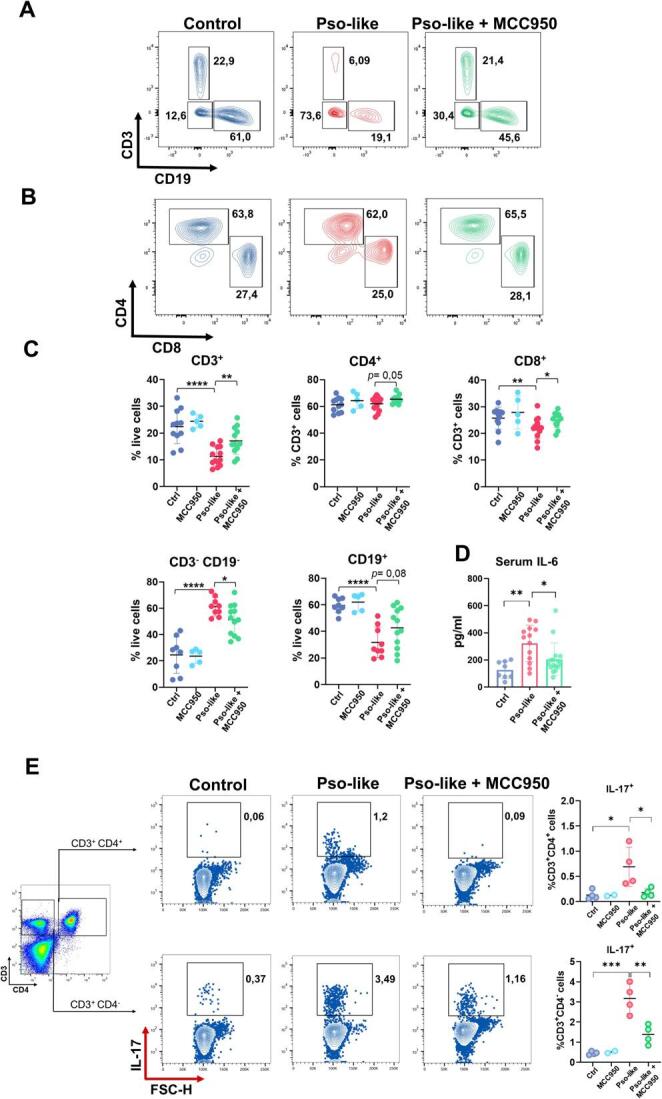
MCC950 pretreatment reverts spleen phenotypic variations and restores IL-6 serum levels in mice with psoriasis-like inflammation. (**A**) Representative FACS plots showing the percentage of CD3⁺ T cells, CD19⁺ B cells, CD3⁻CD19⁻ cells isolated from spleens of control (ctrl), psoriasis-like (pso-like), psoriasis-like MCC950 pre-treated (pso-like+MCC950) mice. Live single cells were selected based on FSC/SSC parameters. (**B**) Representative FACS plots show the percentage of CD4^+^ and CD8^+^ T cells, within CD3^+^ T cells compartment gated in live splenocytes. (**C**) Dot plots depict the percentage of total CD3⁺, CD3⁺CD4⁺, and CD3⁺CD8⁺ populations among viable singlets (ctrl, *n* = 11; MCC950, *n* = 5; pso-like, *n* = 13; pso-like+MCC950, *n* = 12), CD19⁺ and CD3⁻CD19⁻ populations (ctrl, *n* = 8; MCC950, *n* = 5; pso-like, *n* = 9; pso-like+MCC950, *n* = 12) calculated within viable singlet cells. (**D**) Bar graph showing IL-6 concentration (pg/mL) comprising both pre- and post- imiquimod administration in control (ctrl, *n* = 8), psoriasis-like (pso-like, *n* = 13), psoriasis-like MCC950 pre-treated (pso-like+MCC950, *n* = 16) mice. Data is presented as mean ± SD. (**E**) Splenocytes from each mouse were stimulated with PMA 50 ng/ml and ionomycin 500 ng/ml in the presence of Brefeldin A 5 µg/ml for 4 h and intracellularly stained for IL-17 A detection. Representative FACS plots showing IL-17 producing cells within CD3⁺CD4⁺ and CD3⁺CD4⁻ T cell compartment from control (ctrl), psoriasis-like (pso-like), and psoriasis-like MCC950 pre-treated (pso-like + MCC950) mice. Live single cells were gated based on FSC/SSC parameters. Dot plots show the percentage of IL-17⁺ cells within the CD3⁺CD4⁺ and CD3⁺CD4⁻ T cell compartments among viable singlets (ctrl, *n* = 4; MCC950, *n* = 2; pso-like, *n* = 4; pso-like + MCC950, *n* = 4). Data are presented as mean ± SD. Statistical significance: *p < 0.05, **p < 0.01, ***p < 0.001, ****p < 0.0001

Analysis of total CD3⁺ T cells revealed a significant systemic effect of psoriasis-like inflammation. Mice exhibited a significant reduction in the percentage of splenic CD3⁺ T cells, which was fully prevented by topical MCC950, restoring levels comparable to control group (Fig. 5A, C). When analyzing CD4⁺ T helper cells, no statistically significant differences were observed across the groups. CD8^+^ T cells showed a decrease percentage in mice with psoriasis-like inflammation relative to controls and MCC950 pretreatment prevented this reduction (Fig. 5B, C).

We examined the CD3⁻CD19⁻ compartment, a heterogeneous population of innate immune cells. Induction of psoriasis-like inflammation resulted in a strong increase in CD3⁻CD19⁻ cells relative to controls, indicating systemic inflammation-driven increase of innate immune cells. MCC950 pretreatment significantly reduced this expansion (Fig. 5A, C). Data also showed increased frequency of CD11b⁺ cells in the spleen in psoriasis-like mice, which was partially normalized by MCC950, whereas CD11c⁺ cells remained unchanged (Supplemental Fig. 3). Notably, intracellular cytokine staining (ICS) revealed a marked increase in IL-17 A production within both the CD3^+^CD4^+^ and CD3^+^CD4^−^compartments in imiquimod-treated mice. MCC950 skin administration effectively reduced the accumulation of IL-17-producing T cells in the spleen (Fig. 5E). The main IL-17 A producers resulted CD3^+^CD4^−^, likely encompassing γδ T cells [32].

Alongside, at systemic level, serum IL-6 was significantly increased in psoriasis-like condition and was reduced by MCC950 skin treatment (comprising both pre- and post- imiquimod administration groups) (Fig. 5D). Therefore, the decrease of IL-6 in the serum and the parallel reduction of IL-17A^+^ T cells by MCC950 importantly indicate an overall reduction of systemic inflammation.

## Discussion

The results of this study indicate that inhibiting the inflammasome sensor molecule NLRP3 by cutaneous administration of MCC950 can effectively prevent clinical signs of psoriasis-like inflammation. Local NLRP3 inhibition also prevents the first systemic manifestation, which is weight loss and preserved skin tissue architecture during imiquimod treatment. Acanthosis and parakeratosis, which are the histological hallmarks of both the human disease and the mouse model pathology, were completely prevented and there was a marked reduction in immune cell infiltration.

The reduction in epidermal thickness and inflammatory cell infiltration observed in MCC950-treated mice, reinforces the evidence that NLRP3 inflammasome plays a key role in the early phase of psoriasis in this model. Inhibiting NLRP3 by MCC950 may interrupt the translation of epidermal stress signals to dendritic cells that are central for the formation of DC: T cell clusters [33–35]. Therefore, early disruption of inflammasome-mediated cytokine release may have profound effects on both epidermal remodeling and immune response patterns.

Immunofluorescence staining revealed a reciprocal redistribution of T cells between the skin and the spleen under psoriasis-like conditions. This immune cell redistribution was effectively prevented by topical MCC950 administration. Skin recruitment of CD8^+^ T cells likely favors activation which in turn may lead to the shift towards the T_RM_ phenotype [7, 36–42].

Gene expression analysis in the skin showed a marked reduction in the expression of psoriasis hallmark genes including *Il17a*, *Tnf*,* S100a8/a9* and *Il1b*, upon MCC950 treatment.

Western Blot analysis revealed inhibition of caspase-1 activation suggesting a reduction of IL-1β maturation that in turn could limit downstream differentiation of Th17/Tc17 cells and IL17A production [20, 43].

Expression of *Aim2* and *Nlrp3*, which were increased in psoriasis-like conditions, was also reduced by MCC950. The results suggest that topical NLRP3 inhibition reduces key pathogenic cytokine signals at the transcriptional level and may also be linked to AIM2 inflammasome expression [44, 45].

STRING analysis showed a strong correlation with our real-time qPCR findings. NLRP3 was identified as a key hub, directly interacting with seven validated partners. Collectively, data indicates that targeting the single hub NLRP3 can dampen the psoriasis-associated module.

Finally, we found an increase of serum level of IL-6 in psoriasis-like conditions and a normalization induced by MCC950 treatment. IL-6 acts as a key regulator linking epidermal immune dysregulation to systemic inflammation [46, 47]. Accordingly, at systemic level, we also evidenced reduced spleen accumulation of IL-17 A producing cells in mice treated with MCC950 thus strongly reinforcing the potential of upstream inhibition of the inflammatory cascade.

Together, our results emphasize the role of the inflammasome complex in the initial phase of psoriasis and indicate a link with systemic inflammation associated with the disease. This will open new perspectives for upstream targeted therapeutic interventions limiting systemic drug administration with the capability to achieve sustained remission both at cutaneous and at systemic level.

## Materials and Methods

### Mice

All experimental procedures involving animals were conducted in accordance with the EU Directive 2010/63/EU for animal experiments. The entire project was approved by the Italian Ministry of Health (Aut. n.1067/2023-PR and n. 171/2026-PR).

Male and Female C57BL/6 mice aged between 6 and 12 weeks were bred at the Laboratory of Preclinical Research (LARP) of the University of Ferrara and maintained in pathogen-free conditions inside individual ventilated cages. The temperature and humidity remain stable with a 12-hour light/dark cycle. Animals received rodent chow and water *ad libitum*.

### Induction of Psoriasiform Dermatitis and Topical Administration of MCC950

Psoriasiform skin inflammation was induced through topical application of imiquimod. Mice were anesthetized with 2% isoflurane, and the dorsal skin and ears were carefully shaved. Imiquimod cream (Aldara 5%, 50 mg/day) was then applied uniformly to the shaved skin areas and both ears.

Imiquimod was applied daily for 6 consecutive days. Animals assigned to this protocol were euthanized on day 8 to evaluate short-term inflammatory responses [28].

In treatment groups, MCC950 (Sigma-Aldrich) was prepared freshly at a concentration of 1 mg/mL in Milli-Q water and applied topically (200 µL/area) to the same skin areas two hours prior to each imiquimod application. The topical concentration of MCC950 (1 mg/mL) was selected based on the previously reported studies using locally administered (patent application WO 2017/129897 A1), or systemic NLRP3 inhibitors [48].

A reverse treatment schedule was adopted to assess whether MCC950 efficacy was independent of treatment order. In this protocol, imiquimod was applied first, and residual cream was removed before topical administration of MCC950 (1 mg/mL in Milli-Q water; 200 µL/area) after a 12-hour interval. This procedure was repeated daily for 4 consecutive days.

Animals were euthanized on day 4 in the afternoon, and skin, spleen and serum were collected for downstream histological, molecular and immunological analyses. For each experimental group, at least 7 mice were used, except for the control group treated only with MCC950.

### Clinical Assessment

Mice were monitored daily for clinical signs of skin inflammation. A modified Psoriasis Area and Severity Index (PASI) scoring system was used to measure disease severity, recording erythema, scaling, and skin thickening individually on a scale from 0 (none) to 4 (very severe). The cumulative clinical score in each mouse at each time point, was given by the sum of the three parameters. Body weight was measured daily and expressed as the percentage change from baseline (day 1) to day 6 to monitor general health status and systemic effects of the treatment [49].

### Tissue Collection

At the predetermined endpoints, mice were euthanized by cervical dislocation. Dorsal and ear skin samples were excised for histological analysis, immunofluorescence studies, and molecular assays. Additionally, spleens were harvested and processed for immunophenotyping by flow cytometry, and blood samples were collected via submandibular venipuncture for subsequent serum cytokine analysis. All collected tissues were either fixed immediately or snap-frozen in liquid nitrogen and stored at − 80 °C until use [49].

### Histological Analysis

For histopathological evaluation, skin biopsies were fixed in 4% paraformaldehyde for at least 24 h, processed, and embedded in O.C.T compound (Histo-Line Laboratories Milan, Italy). 7 μm sections were cut using a cryostat and stained with hematoxylin and eosin (H&E) [50]. Morphological features were assessed by light microscopy, including measurement of epidermal thickness, which was quantified in micrometers at 2 randomly selected fields per section using digital imaging software ImageJ. Representative images were analyzed with a Nikon D-FL-E model LH-M100C-1 Digital sight DS-2Mv at 10x and 20x magnification.

### Immunoblot Analysis

Total protein was extracted from skin samples using RIPA buffer added with phosphatase and protease inhibitors. Protein concentrations were quantified with a BCA Protein Assay Kit (Thermo Fisher Scientific, Waltham, MA, USA). Protein extracts obtained from skin samples of imiquimod-treated mice pre-treated with MCC950 (0.2 mg/mL or 1 mg/mL), together with untreated control mice were separated on 12,5% polyacrylamide denaturing gels and blotted onto nitrocellulose membranes (GE Healthcare Life Science, Little Chalfont, UK). The membranes were then incubated with antibodies directed against Pro-caspase-1 (#83383; Cell Signalling Technology, Danvers, MA, USA), Caspase-1 p20 (A87644; Antibodies.com, Stockholm, Sweden) and β-Actin (#A4700, Merck KgaA), following manufacturers’ instruction, incubated with peroxidase-conjugated secondary antibodies and revealed with the ECL system (Cyanagen, Bologna, Italy).

The chemiluminescence of the resolved bands was acquired with an ImageQuantTM LAS 4000 biomolecular imager (GE Healthcare Life Science), and densitometric analysis was performed by Image Quant TL software (GE Healthcare Life Science).

### Direct Immunofluorescence (dIF) Staining

Back skin samples were obtained from control mice, imiquimod (IMQ)-treated psoriasiform mice, and mice with psoriasis-like inflammation pretreated with MCC950. Frozen back skin Sect.  (7 μm) were prepared on Superfrost Plus slides and stored at − 20 °C until the use. After air drying, sections were blocked to prevent nonspecific binding. For CD3-FITC (1:100, cat. no. 130-119-798, Miltenyi, Bologna, Italy, IT), CD11b-PE (1:100, cat. no. 130-113-806, anti-mouse REA592),CD11c-PE (1:100, cat. no. 130-110-838, anti-mouse REA592), staining, sections were incubated with 10% BSA in PBS. For CD4-PE (1:50, cat. no. 130-116-509, anti-mouse REA604), CD8a-FITC (1:50, cat. no. 130-118-468, anti-mouse 53 − 6.7) staining, sections were blocked with 5% FCS and 0.1% Tween-20 in PBS. All antibodies were diluted in the corresponding blocking solution and incubated overnight at 4 °C in the dark. Following incubation, sections were washed with 0.04% Tween-20 in PBS for 1 min before nuclear staining. Nuclei were counterstained with DAPI 4’,6-diamidino-2-phenylindole (cat.no. 62248 Invitrogen™) for 15 min. Slides were then mounted with Fluoromount-G™ Mounting Medium (cat.no. 00-4958-02 Invitrogen™) and analyzed using a direct fluorescence microscope Nikon Ci-L microscope (Nikon) equipped with a DS-Qi2Mc digital camera (Nikon). Fluorescent pictures were captured by NIS-Elements BR Imaging Software (Nikon). Images were captured and processed using Image-J software, and CD3^+^, CD11b^+^, CD11c^+^, CD4^+^ and CD8^+^ cell densities were quantified within defined regions of interest (ROIs). Interfollicular signals were systematically excluded from quantitative analysis.

At least 4 mice per group were analyzed, and for each mouse, two independent cryosections from different skin areas were examined, and for each section, two different ROIs were analyzed.

### RNA Extraction and RT-qPCR

Total RNA was extracted from skin tissue using TRIzol™ Reagent (ThermoFisher™, Milan, Italy).

following the producer’s instruction and purified it with RNeasy Mini Kit (Qiagen™, Milan, Italy). The concentration of RNA was determined by spectrophotometer by measuring absorbance at 260/280 nm. Reverse transcription was performed using SuperScript™ III Reverse Transcriptase (ThermoFisher™ Milan, Italy) to produce complementary DNA (cDNA). Quantitative real-time PCR (qPCR) was performed using PerfeCTa SYBR^®^ Green SuperMix ROX Reagent (QuantaBio). Specific primers (Supplemental Table 1) (Sigma Aldrich™ Milan, Italy) were used. The relative expression of the genes was calculated using the 2^−ΔCT and normalized using housekeeping gene *Gapdh*.

### In Silico Gene Expression and Network-based Analysis

Computational analysis of human psoriasis transcriptomic data (dataset GSE54456) was performed focusing on inflammasome components, cytokines, and other psoriasis-associated markers [29]. Data was re-processed using R software to extract fold-change and RPKM values for graphical representation. Interaction networks and co-expression values were retrieved from the STRING database (version 12.0) (confidence > 0.9) [51]. Scores represent Pearson correlation coefficients calculated across more than 300 uniformly re-processed human RNA-seq and proteomics datasets from public repositories (Expression Atlas and ProteomeHD) integrated within STRING v12.0. Only correlations supported by at least 10 paired measurements were retained. Human data included 486 bulk RNA-seq and 312 LC-MS proteomics runs uniformly re-processed by the EMBL-EBI Expression Atlas. STRING v12.0 network results were cross-validated with experimental and biochemical evidence, including co-precipitation assays [31] and yeast two-hybrid (Y2H) data [30].

### Serum Cytokine Analysis

Mouse serum was collected from mice on day 3 during the imiquimod treatment. Serum was diluted from 1:3 to 1:10 in serum dilution buffer and quantified with IL-6 Elisa Kit (PeproTech *cat. n.*900-T50) according to the manufacturer’s instruction. Samples were rum duplicate, measuring the absorbance at 450 nm with a microplate reader. Cytokine concentrations were determined by comparing them with standard curves generated at known concentrations.

### Flow Cytometry

Single-cell suspensions were prepared from harvested spleens by mechanical separation through a 70 μm cell strainer in phosphate-buffered saline (PBS). Red blood cells were lysed using 1X lysis buffer (composed by 8.26 g NH4Cl, 1 g KHCO3, and 0.037 g EDTA in 100 mL Milli-Q water). After washing and counting, cells were incubated with fluorochrome-conjugated antibodies against CD3 (cat.no.130-120-826, PerCP-Vio^®^ 700 anti-mouse REA641), CD4 (cat.no. 130-118-692, Vio^®^ Bright FITC anti-mouse REA604), CD8 (cat.no. 130-123-865, VioBlue, anti-mouse 53 − 6.7) and CD19 (cat.no. 130-112-037, PE-Vio770, anti-mouse REA592) (Miltenyi Biotech GmbH, Bergisch Gladbach, Germany), CD11b-PE (cat. no. 130-113-806, anti-mouse REA592) and CD11c PE (cat. no. 130-110-838, anti-mouse REA592). Viability was assessed using LIVE/DEAD™ Fixable Aqua Dead Cell Stain Kit (Invitrogen). Data acquisition was performed on a FACSAria III flow cytometer (BD Biosciences, Franklin Lakes, NJ, USA), and data were analyzed with FlowJo software v10.10 (BD Bioscience). The gating strategy is reported in Supplemental Fig. 1. Gating strategies were defined based on fluorescence minus one (FMO) control.

### Intracellular IL-17 A Detection by Flow Cytometry in Splenocytes

Splenocytes were cultured in RPMI-1640 medium supplemented with 10% fetal bovine serum, 100 U/mL penicillin, and 100 µg/mL streptomycin at 37 °C in a 5% CO₂ atmosphere. The cells were seeded in 24-well plates at a density of 3 × 10^6^ cells/mL (1 mL per each well) and stimulated for 4 h. with 50 ng/mL of Phorbol 12-myristate 13-acetate (PMA), 500 ng/mL of ionomycin in the presence of 5 µg/mL of Brefeldin A (BFA).

After incubation, cells were washed in FACS buffer and stained for 15 min at 4 °C for surface markers (CD3-PerCP-Vio^®^ 700, Milteniy, CD4-VioBlue, Milteniy, LIVE/DEAD™ Fixable Aqua Dead Cell Stain Kit (Invitrogen). For intracellular staining, the cells were washed in FACS buffer, permeabilized using the Cytofix/Cytoperm Kit (BD Biosciences), and stained for 20 min at 4 °C with IL-17 A-FITC (Miltenyi). Between each step, cells were washed twice with cold FACS buffer. Data acquisition was performed on a FACSAria III flow cytometer (BD Biosciences, Franklin Lakes, NJ, USA), and data were analyzed with FlowJo software v10.10 (BD Bioscience).

### Statistical Analysis

Statistical comparisons between groups were performed using unpaired Student’s t-test for normally distributed data or the Mann-Whitney U test for non-normally distributed data, as determined by the Shapiro-Wilk normality test. Statistical analyses were conducted using GraphPad Prism software (GraphPad, La Jolla, CA, USA). Differences were considered statistically significant at p-values ≤ 0.05.

## Supplementary Information

Below is the link to the electronic supplementary material.

Supplementary Material 1

## Data Availability

All raw data in this study, including raw RT-qPCR values, flow-cytometry analysis (FCS), immunofluorescence picture, and histological data, are available from the corresponding author upon request.Human transcriptomic data used were obtained from an available Gene Expression Omnibus dataset GSE54456. The dataset is not associated with a registered clinical trial number.The dataset is available at: [https://www.ncbi.nlm.nih.gov/geo/query/acc.cgi? acc=GSE54456](https:/www.ncbi.nlm.nih.gov/geo/query/acc.cgi? acc=GSE54456)Data citation (DataCite-compliant): Transcriptome analysis of psoriasis in a large case-control sample: RNA-seq provides insights into disease mechanisms. Gene Expression Omnibus, GSE54456. [https://identifiers.org/geo: GSE54456](https:/identifiers.org/geo: GSE54456) Li B, Tsoi LC, Swindell WR, Gudjonsson JE, Tejasvi T, Johnston A, et al. Transcriptome analysis of psoriasis in a large case-control sample: RNA-seq provides insights into disease mechanisms. J Invest Dermatol.2014;134(7):1828-38. [https://doi.org/10.1038/jid.2014.28](https:/doi.org/10.1038/jid.2014.28) .

## Declarations

### Clinical Trial Number

No clinical trial was conducted as part of this research. Therefore, clinical trial number: not applicable.
