# Supplementary material for: Skin Application of Inflammasome Inhibitor MCC950 Prevents Psoriasis-like Cutaneous Manifestations and Associated Systemic Inflammation
Source: Inflammation. 2026 Jun 4;49(1):173. doi: 10.1007/s10753-026-02535-1 (PMC13453980; doi:10.1007/s10753-026-02535-1)
Supplement: Supplementary file 1 [file 10753_2026_2535_MOESM1_ESM.pdf]

Skin Application of Inflammasome Inhibitor MCC950 prevents Psoriasis-Like Skin Manifestations and Associated Systemic Inflammation

*[1]Chiara Terranova, *[2]Alex Spitilli, *[1]Alisia Camporeale, [1]Francesca Nigro, [1]Federica Brugnoli, [3]Silvia Grassilli, [1]Valeria Bertagnolo, [3]Fabio Casciano, [4]Hasan Ayaz,
[3]Enrico Tassani [5]Chiara Ruzza, [6]Claudio Trapella,[4]Francesca Granucci,
[2]Davide Ferrari, [1]Paola Secchiero and [1§]Eva Reali

*The authors equally contributed to this work

[1]Department of Translational Medicine, University of Ferrara, Ferrara, Italy

[2]Department of Life Sciences and Biotechnology, University of Ferrara, Ferrara, Italy

[3]Department of Environmental and Prevention Sciences and LTTA Centre, University of Ferrara, Ferrara, Italy

[4]Department of Biotechnology and Biosciences, University of Milano-Bicocca, Milan, Italy

[5]Department of Neurosciences and Rehabilitation, University of Ferrara, Ferrara, Italy

[6]Department of Chemical Pharmaceutical and Agricultural Sciences, University of Ferrara, Ferrara, Italy

Keywords: NLRP3 inflammasome, Skin Inflammation, Upstream targeting, T-cell trafficking, Systemic effects.

§Address for Correspondence:
Eva Reali, Department of Translational Medicine, University of Ferrara, Ferrara, Italy
Email: eva.reali@unife.it

Supplementary Information

Supplemental Figure 1

Gating Strategy

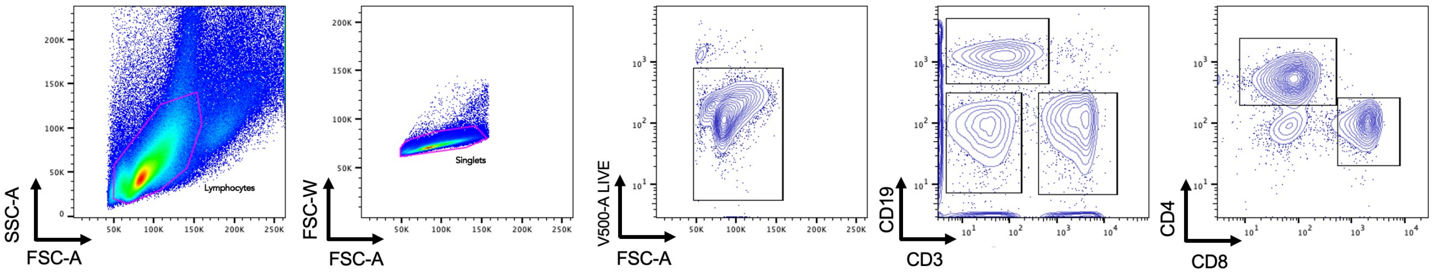

Supp Fig.1

Representative gating strategy used to identify $CD3^+$ T, $CD19^+$ B and double negative cells. After excluding doublets and dead cells, and selection of live cells. The panels indicate the gating procedures used for $CD3^+$ T cells and their subtypes, $CD4^+$ and $CD8^+$, $CD19^+$ cells and $CD3^-CD19^-$ cells.

Supplemental Figure 2

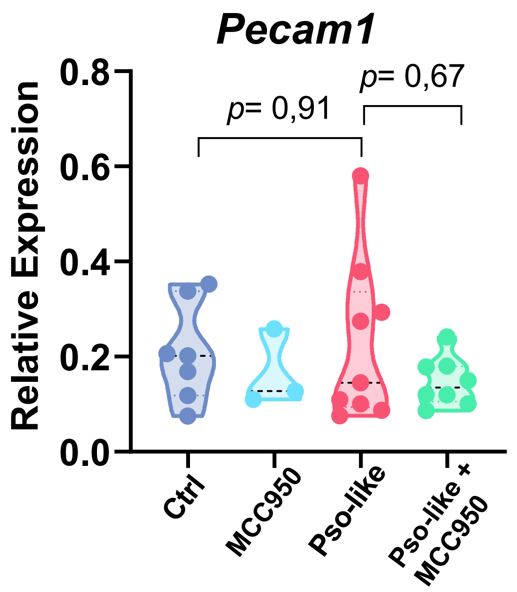

Supp Fig.2

Gene expression analysis by qRT-PCR was performed in skin samples from control (ctrl, n = 7), MCC950 alone (MCC950, n = 3) psoriasis-like mice (pso-like, n = 9) and psoriasis-like MCC950 pre-treated (pso-like + MCC950, n = 8). Relative expressions were calculated using the 2−DCT formula and *Gapdh* as a reference gene. Violin plot showing the relative expression for *Pecam1* gene in different conditions is reported in the figure. Statistical analysis of the differences was performed by Mann–Whitney test or Student t-test according to the normality of the distribution. All data were considered statistically significant at *p < 0.05, **p < 0.01, ***p < 0.001, ****p < 0.0001.

Supplemental Figure 3

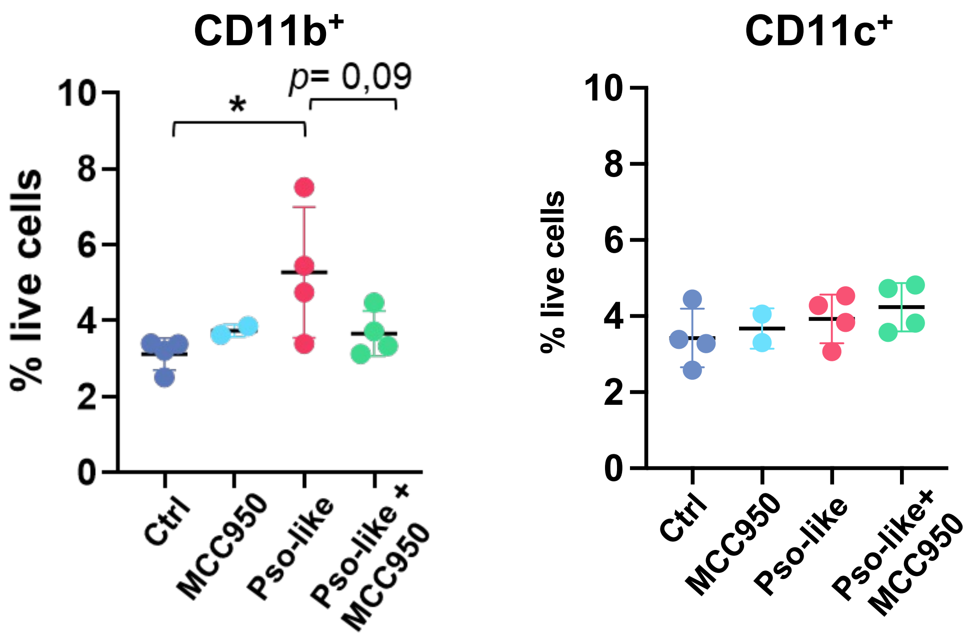

Supp Fig.3

Dot plots depict the percentage of total CD11b+ and CD11c+ populations (ctrl, n=4; MCC950, n=2; pso-like, n=4; pso-like+MCC950, n=4), calculated within viable singlet cells. Data are presented as mean ± SD. Statistical significance: *p < 0.05, **p < 0.01, ***p < 0.001, ****p < 0.0001.

Supplemental Table 1

Primer name	Primer Sequence
Gapdh – F	ACCACAGTCCATGCCATCAC
Gapdh – R	TCCACCACCCTGTTGCTGTA
Il17 – F	CAGACTACCTCAACCGTTCCAC
Il17 – R	TCCAGCTTTCCCTCCGCATTGA
Tnf – F	GCACAGAAAGCATGACCCG
Tnf – R	GCCCCCCATCTTTTGGG
Il1β - F	TGCCACCTTTTGACAGTGATG
Il1β - R	ATGTGCTGCTGCGAGATTTG
Il22 – F	GCTTGAGGTGTCCAACTTCCAG
Il22 – R	ACTCCTCGGAACAGTTTCTCCC
S100a8 – F	CAAGGAAATCACCATGCCCTCTA
S100a8 – R	ACCATCGCAAGGAACTCCTCGA
S100a9 – F	TGGTGGAAGCACAGTTGGCAAC
S100a9 – R	CAGCATCATACACTCCTCAAAGC
Nlrp3 – F	TCACAACTCGCCCAAGGAGGAA
Nlrp3 – R	AAGAGACCACGGCAGAAGCTAG
Aim2 – F	AGGCTGCTACAGAAGTCTGTCC
Aim2 – R	TCAGCACCGTGACAACAAGTGG
Pecam1 – F	CCAAAGCCAGTAGCATCATGGTC
Pecam1 – R	GGATGGTGAAGTTGGCTACAGG

Supplemental Table 1. Primers
